# From good to great: learners’ perceptions of the qualities of effective medical teachers and clinical supervisors in psychiatry

**Published:** 2019-07-24

**Authors:** Sheila Harms, Bryce J. M. Bogie, Anne Lizius, Karen Saperson, Susan M. Jack, Meghan M. McConnell

**Affiliations:** 1 Michael G. DeGroote School of Medicine, McMaster University, Ontario, Canada; 2 Department of Psychiatry and Behavioural Neurosciences, McMaster University, Ontario, Canada; 3 Neuroscience Graduate Program, McMaster University, Ontario, Canada; 4 School of Nursing, McMaster University, Ontario, Canada; 5 Department of Innovation in Medical Education, University of Ottawa, Ontario, Canada; 6 Department of Anesthesiology and Pain Medicine, University of Ottawa, Ontario, Canada

## Abstract

**Background:**

The shift in postgraduate medical training towards a competency-based medical education framework has inspired research focused on medical educator competencies. This research has rarely considered the importance of the learning environment in terms of both setting and specialty-specific factors. The current study attempted to fill this gap by examining narrative comments from psychiatry faculty evaluations to understand learners’ perceptions of educator effectiveness.

**Methods:**

Data consisted of psychiatry faculty evaluations completed in 2015-2016 by undergraduate and postgraduate learners (*N* = 324) from McMaster University. Evaluations were provided for medical teachers and clinical supervisors in classroom and clinical settings. Narrative comments were analyzed using descriptive qualitative methodology by three independent reviewers to answer: “What do undergraduate and postgraduate medical learners perceive about educator effectiveness in psychiatry?”

**Results:**

Narrative comments were provided on 270/324 (83%) faculty evaluation forms. Four themes and two sub- themes emerged from the qualitative analysis. Effective psychiatry educators demonstrated specific personal characteristics that aligned with previous research on educator effectiveness. Novel themes included the importance of relationships and affective factors, including learner security and inspiration through role modeling

**Conclusion:**

Contemporary discussions about educator effectiveness in psychiatry have excluded the dynamic, relational and affective components of the educational exchange highlighted in the current study. This may be an important focus for future educational research.

## Introduction

Competency-based medical education (CBME) is an outcome-based, learner-centered approach to medical education.^1–3^ CBME emphasizes incremental attainment and mastery of abilities, or *competencies*, as learners progress through medical training towards full competence as an independent physician.^1-4^ In order for CBME frameworks to be effective in producing competent physicians, medical teachers and clinical supervisors must also be competent in their teaching and supervisory abilities.^1,3^

Unlike the standards of competence required of physicians, medical teachers and clinical supervisors are not required to demonstrate mastery of teaching and supervisory competencies before adopting their respective roles as medical educators.^5,6^ This has led to a call for training and faculty development programs for all medical educators.^5-11^ Previous faculty development programs have focused on training medical educators according to general teaching strategies applicable to all medical specialties.^9,12,13^ For example, Dewey and colleagues recently proposed a framework that focuses on assessing medical educators’ competence on educational entrustable professional activities.^9,14^ Consistent with this framework, O’Donovan and colleagues piloted a two-day general clinical supervision training program that included a combination of: large group lecture-type learning, observational learning, role-play, and corrective feedback from participants’ recorded supervision sessions.^12^ Participants demonstrated a statistically significant improvement in evaluator-rated supervisor competence following this program.

Research investigating the characteristics of effective medical educators has also taken a predominantly non-specialty-specific approach. For instance, in their comprehensive systematic review, Sutkin and colleagues analyzed the literature between 1909-2006 and identified 49 themes that represented good medical teachers, which they organized into three categories: physician, teacher, and human characteristics.^15^ These themes were characterized as either cognitive (i.e., involving clinical perceptions) or non-cognitive (i.e., involving personal qualities and interpersonal skills) characteristics. Interestingly, 67% of the identified themes described non-cognitive characteristics of educators as being salient. Examples of non-cognitive characteristics included communication skills, enthusiasm, and compassion/empathy. More recently, Srinivasan and colleagues developed a *Teaching as a Competency* framework.^16^ Six core competencies for all medical educators were incorporated into this framework: medical knowledge, learner-centeredness, interpersonal/communication skills, professionalism, practice-based reflection, and systems-based practice. These researchers recommended that each competency was “definitely needed” for all clinical teachers, individual or small group teachers, and large group teachers. Moreover, Stenfors-Hayes and colleagues conducted interviews with medical teachers and clinical supervisors to probe the question of what makes a good medical teacher and clinical supervisor; results are summarized in Table 1.^17^ Similarities between these three *general* frameworks for effective medical educators are evident. For example, note the emphasis on effective communication skills within each framework.

**Table 1:** Hierarchical summary of the factors that define good medical teachers and clinical supervisors from Stenfors-Hayes and colleagues’ phenomenographic analysis^17^

Good Medical Teachers	Good Clinical Supervisors
Support students’ learning	Promote learners’ development
Respond to students’ content requests	Are role models and shareclinical experiences
Are knowledgeable and skilled in their content area	Teach within clinical cases

While results from previous research have suggested that general training may improve the competence of medical educators, questions remain about the effectiveness of such generalized programs across different medical specialties. For example, it remains unclear whether medical educators in different medical specialties require the same educator competencies. Research in psychiatry has begun to focus efforts on identifying specific educator competencies. For example, an understanding of what constitutes beneficial feedback from the psychiatry learner's perspective was studied by Telio and colleagues within the context of an educational alliance framework.^18^

The contexts in which medical teaching and clinical supervision occur in psychiatry are unique. For example, medical teaching traditionally occurs in the classroom with many learners and is structured and planned, whereas clinical supervision occurs in a clinical environment with one or a few learners, and typically involves spontaneous, less structured bedside teaching.^5,17^ This distinction is important because many different conceptions of teaching have been identified, each being more or less applicable in different contexts.^19^ How educators conceptualize their teaching roles may directly affect how they teach and, by extension, the quality of teaching their learners receive.^19^ Therefore, given the contextual differences between medical teaching and clinical supervision, and considering the large variability in clinical environments and situations across medical specialties, it is conceivable that differences exist between the qualities that make a good medical teacher and clinical supervisor across specialties. While previous literature has identified qualities of good medical educators *in general*, it remains to be seen whether different medical specialties require different competencies for their educators.

To help fill this knowledge gap, we aimed to examine narrative comments from psychiatry faculty evaluations to understand learners’ perceptions of educator effectiveness within psychiatry. The primary objective of this research was therefore to identify qualities of effective medical teachers and clinical supervisors within psychiatry as reported by medical students and residents. Given that faculty evaluation forms were designed to capture feedback on educator performance, these forms provided a large and rich data set generated by high-level learners which reflected educator competence. For the purpose of this paper, the term “educator” refers to both medical teachers and clinical supervisors, unless otherwise indicated.

## Methods

### Study design

The overarching research question for this study was: “What do undergraduate and postgraduate medical learners perceive about educator effectiveness in psychiatry?” Using existing narrative comments from faculty evaluations, a fundamental qualitative descriptive design was selected because it was most appropriate given the available data. The principles of qualitative description guided all decisions related to sampling, data collection, analysis, and interpretation. This method also aligns best when the data occur in a real-life context.^20^ The ethics approval protocols for McMaster University and the Hamilton Integrated Research Ethics Board were followed and approved (#11-409) with the explicit understanding that narrative evaluation data completed by learners about faculty would be analyzed. Both undergraduate and postgraduate medical learners at McMaster University receive multiple communications that the completion of formal evaluations are important for programmatic change and faculty development, thereby alerting learners to the possibility of using evaluation data for these purposes. All faculty evaluations are routinely collated to create an evaluation summary which is then shared with the faculty member. Evaluation summaries include all narrative comments and mean scores, although learner anonymity is preserved. A robust process exists to address articulated concerns about faculty performance within McMaster University’s Department of Psychiatry and Behavioural Neurosciences.

### Setting and data collection

This study was conducted at McMaster University in the Department of Psychiatry and Behavioural Neurosciences. Canadian accreditation standards require written evaluations for all educators to ensure a robust academic environment. At McMaster University, educator evaluation forms include opportunities for qualitative comments and quantitative ratings of educator performance. The context for these evaluations includes didactic teaching sessions and clinical supervision. Teaching encounters consist of small group learning of core curriculum in both undergraduate and postgraduate medical programs. Clinical supervisor evaluations are completed by both undergraduate and postgraduate learners during core rotations and electives across a variety of inpatient and outpatient clinical settings.

All existing psychiatry faculty evaluations from undergraduate and postgraduate learners during the 2015 and 2016 academic cycles (*N* = 324) were included in the analysis. This study used faculty evaluation forms from two different settings: classroom-style and clinical supervision environments. Both quantitative and qualitative data were extracted from all forms. Unfortunately, evaluation forms were not standardized across educational settings and thus different quantitative and qualitative questions were posed. Each evaluation form included several Likert scale items about educator effectiveness. However, these quantitative data were not included in the present study because the Likert scale items were different across evaluation forms in question content and in the number of response items per question (e.g., 5- point versus 7-point Likert scales). Additionally, nearly all responses were high (i.e., positively-biased), making a distinction between good versus poor performance impossible. On the other hand, the different faculty evaluation forms all included open- ended fields allowing for narrative comments. These fields included written prompts such as “areas of strength,” “areas for improvement” and “comments.”

The faculty members represented a continuum of educational and clinical experience, from junior to senior faculty. Within the postgraduate education program at McMaster University, clinical supervisors are selected by psychiatry residents, whereas in undergraduate psychiatry training, medical students are matched with psychiatry faculty through an administrative process. Selection of faculty to teach undergraduate sessions is largely influenced by those who agree to take on these teaching opportunities. Faculty who are selected to teach academic sessions in the postgraduate training program depend on positive evaluations from previous teaching sessions. The research team consisted of individuals with varied professional backgrounds, including educational leadership (SH, KS), expertise in qualitative and quantitative methodology, respectively (SH and SJ, MM), and student involvement (BB, AL).

### Data analysis

All data were completely anonymized by a research assistant who did not participate in data analysis. All data identifiers, such as names, gender, rotation, location, clinical focus, specialty, and professional level were removed to ensure complete anonymity. Data were then extracted by transferring qualitative comments from each faculty evaluation form onto an Excel spreadsheet, categorized broadly by the two focus areas of evaluation (i.e., teaching and supervision).

In keeping with the fundamental qualitative descriptive study design, the researchers articulated assumptions and personal biases prior to data analysis through a process of journaling. Throughout the coding process, the authors also engaged in reflexive practice by keeping journals as a way of understanding and documenting personal assumptions, biases, and beliefs about medical education in psychiatry. Reflexivity in qualitative research was a process used to establish rigor and promote credibility of the data by ensuring that researchers: carefully examined their roles in the generation of knowledge, recognized the potential influence of their experiences on data interpretations, and ensured that final interpretations reflected the participants’ reported experiences.^21^

Faculty evaluation narratives were analyzed in two separate groups: medical teaching and clinical supervision. The computer software package QSR NVivo 11 for Windows Starter Edition (https://www.qsrinternational.com/nvivo/home) was used in data management and coding. Techniques associated with conventional content analysis were applied to code and categorize the narrative comments. Narrative comments were independently coded by three researchers (SH, BB, AL) until saturation of themes emerged.^22^ Anonymity of the data ensured that there were no conflicts of interest among coders regarding their interpretation of the narrative comments. Following the reading of all entries, first-level coding was conducted to identify key phrases in the text, using the research question as a guide. Codes were subsequently assigned to these key phrases.^23^ Second-level coding was then completed to identify overarching categories and to establish the relationships and links across these categories.^24^ Following data reduction, key themes were developed through a process of interpretation of the narrative comments. Assumptions made by researchers prior to data analysis were frequently referred to throughout coding in order to strengthen confirmability and objectivity.

## Results

From the 324 forms, 270 (83%) included narrative data that were extracted. Following data analysis, four main themes and two sub-themes emerged.

### Personal characteristics

Analysis of the current data produced an extensive list of *Personal Characteristics* describing desirable qualities of medical educators in psychiatry. In identifying these qualities, learners’ descriptions of medical teachers and clinical supervisors were similar such that the comments could be thematically combined and organized into a list of broad attributes. The following core personal characteristics emerged:

- Learner-centered
- Supportive
- Engaging
- Good communicator
- Respectful
- Professional

Beyond this list of personal characteristics, three additional themes and two sub-themes emerged from the data analysis.

### Relationships matter

This theme applied to both medical teachers and clinical supervisors. The impact of the educator- learner relationship was commented on in a predominantly positive manner. One learner said, “[the educator] cared a lot about our learning.” Another said, “[the educator was] very enthusiastic and [was] invested in making this a great experience for residents.” Finally, another learner commented specifically on the affective experience of the educational relationship by noting that the educator “really brought down [their] level of anxiety.” In each of these comments, the educator embodied and enacted the qualities described as an effective educator, thereby creating the conditions for a strong educator-learner relationship to form. Learners commented that the relationship needs to attend to the specific moments of learning “in a way which is not disruptive to the flow,” thus supporting the learner in their clinical work in a manner that “fosters personal growth and cheerleads [the learner] to be better.”

While the majority of comments about the educator- learner relationship and its impact on the learning experience were positive, there were a few negative examples. One respondent commented:

*[The educator] at times inadvertently discourage[d] learners from contributing to discussions; seem[ed] to have an ‘a priori’ thesis for the discussion and ideas that differ[ed] from this [were] not very welcome. Information was WAY too complex, material was way above me. Did not align with the principles of adult education. Felt odd how people were picked on to give answers*.

When the educator-learner relationship “inadvertently discourage[d] learners from contributing,” it was felt to “not align with the principles of adult education.” This experience was in contrast to the supportive relationship generally described by the majority of learners. This connotes a sense of being belittled and devalued, suggesting again the importance of the relationship that is central to the learning experience.

The narrative comments focused on the affective experience of the learner, as opposed to specific learning outcomes. At the extreme, one learner explained that “by the end of the elective [they] felt quite beaten up; [they were] reduced to tears twice during the elective.” This suggests a total breakdown of the relationship between educator and learner, raising the question as to whether learning had broken down as well.

The learners’ comments also spoke to the relationship as a pivotal learning experience, leaving the reader to fill in the gaps about what content was learned. These quotations also spoke to the dynamic interactions between educator and learner, which were predicated on the experience of affect and not directly on comments related to learning. The affective valence of the descriptions of educator- learner relationships spoke to the central importance of educator-learner relationships across educational settings.

This core theme was apparent in sufficient quantity and depth that two sub-themes of *Learner security: The conditions for optimal learning* and *A spectrum of admiration* were also identified. These two sub- themes were relevant only to the clinical supervisor data, likely due to the extended duration in which learners would have been exposed to their clinical supervisor.

### Learner security: the conditions for optimal learning

The sub-theme of *Learner security: The conditions for optimal learning* was described often in quotations attesting to perceived support from the clinical supervisor. One learner commented, “perfect balance of autonomy and support. [Supervisor] always foster[ed my] personal growth and cheerlead[ed me] to be better but always let [me] know that [supervisor was] there if [I] need[ed the supervisor]. Very supportive, offered to come in and assist with caseloads when seemed like high volumes.” The majority of learners acknowledged the tension between “autonomy and support” between the learner and educator, which was necessary to create an engaging learning environment. It was apparent that learners desired a supervisory relationship marked by specific tensions in which they “felt challenged to develop independent diagnoses and plans, [while feeling] like [they] had back-up if needed.” There was an identified necessity for learners to feel they had a sense of independence to practice and develop their clinical skillsets, while also having security derived from the quality and stability of their relationship.

### A spectrum of admiration

The value of a clinical supervisor becoming a role model was frequently referenced and generally described in an affectively-laden manner. A range of intensity was apparent in these comments, from role model or mentor to someone who was idealized. For example, one supervisor was described as “a wonderful mentor who [was] inspiring and encapsulate[d] everything that I strive to become as a psychiatrist,” evoking a sense of the idolization of the supervisor. Another supervisor was described with admiration because of their ability to provide “guidance around developing work-life balance and incorporating the practice of psychiatry into the bigger picture of life and family.” These quotations were entirely of a positive nature, although they varied in magnitude. Similar to the comments on the educator-learner relationship, the language in these comments was significantly laden with affect.

### Person as pedagogy

The theme of *Person as Pedagogy* was identified exclusively within the narrative comments from medical teacher evaluations, suggesting that there are differences in what constitutes educational excellence based on the educational context. This theme highlights the ways in which medical teachers themselves became the method of teaching. Personal characteristics and modes of engagement demonstrated by the teacher were alluded to in creating the pedagogical confines of a positive educational experience. Comments suggested that the medical teacher became an important pedagogical tool, and that excellent education transcended the material being presented through the teacher as an entry to an unexpected type of learning. This type of engagement also implies a deeply inter-other experience, with prominent cultural and contextual factors influencing both teachers and learners alike. The teacher became the site of accessibility for learning, whether this actually happened or not. However, in the comments provided by learners, the teacher’s personhood was conceptualized as an extension of pedagogy, and the learner’s ability to enter into the educational experiences was offered through the teacher as a type of learning portal, bringing life to the learning phenomenon. There was a predominantly positive skew to these data with quotations speaking to how excellent educational techniques cut through potential learning obstacles. For example:

One of the most engaging seminars I have participated in via video conference. This speaks highly to [the teacher’s] ability to facilitate thoughtful discussion, present material and also be conscious of how this transmits over technology. Superb!

In this quotation, a skilled teacher could facilitate an engaged type of learning that appeared to surprise the learner. Specifically, having the ability to capture the learner’s attention and engage the learner was noted to be an important pedagogical strategy. Although there was a strong positive skew to these quotations, there were some outliers which were highly salient. For example, one learner said that “although [the teacher] provide[d] excellent information, [the teacher’s] disorganization and distraction throughout the seminar [made] it difficult to take away meaningful points related to the set objectives for that session.” These comments revealed that even when excellent information was provided in a teaching session, if the teacher was perceived to lack pedagogical skills, the learner floundered despite having transmitted “excellent information.” The “excellent information” was lost in the teacher’s own lostness, thereby pedagogically enacting what is attributed to the teacher. While there were not many examples of “difficult” teachers, what became obvious was that the perceived challenges inherent in the learning experience, and by extension the perceived difficult nature of the teacher, formatively shaped the learner. Although there is an innate tendency and perhaps an unconscious expectation from learners that learning should be positively-valanced, what is not clearly understood is the relative merit of having a difficult learning experience *vis-à-vis* the difficult teacher.

### Supervisors: more than medical experts

Within the clinical supervisor data, the theme of *Supervisors: More Than Medical Experts* emerged. The role of medical expert is defined in the CanMEDS framework as being the central role of the physician, integrating all other roles into the application of knowledge, skill, and professionalism to provide “high-quality and safe patient-centered care.”^25^ This theme suggests that, while medical expertise was necessary in a clinical supervisor, excellent supervisors are skilled beyond this foundation. For example, one learner commented that a supervisor’s “knowledge was clearly excellent, but rarely shared with [them] as a learner.” It is clear from this quotation that knowledge alone is insufficient to deliver effective clinical supervision. Clinical supervisors must also embody skills and qualities that build upon their knowledge base. For example, being a “mentor, role-model, patient advocate and the list goes on.”

As with the other themes, there was a positive skew to the quotations describing the theme of *Supervisors: More Than Medical Experts*. There were, however, examples of pertinent negative outliers. In these examples, learners described a situation wherein, although they may have “enjoy[ed their supervisor’s] demeanor,” the learners sometimes found their supervisor’s “teaching profoundly lacking.” For example, “[the supervisor] would ask a clinical question only sometimes related to a current case, give only negative feedback and offer no teaching moment, instead initiating disappointment and leaving [the learner] to read at home.” This experience illustrates a learner’s inaccessibility to their supervisor’s medical expertise, leaving them feeling “that [they had] missed out on teaching.” Effective clinical supervision must include both medical expertise *and* the demonstration of skills and personal qualities for effective clinical practice. Comments suggested that either of these in isolation may lead to an environment where the learner could feel disappointed and unfulfilled. It is only when the supervisor demonstrates clinical skills and personal qualities along with medical expertise that the perceived conditions for effective clinical supervision can occur.

In summary, a variety of *Personal Characteristics* were identified, which were in keeping with previous literature; however, novel themes were also identified. The theme of *Relationships Matter* emerged in both educational settings, suggesting that the ability to develop some type of educational relationship in psychiatry education is of vital importance irrespective of the learning environment. In the medical teaching context, the theme of *Person as Pedagogy* was identified to represent ways in which the medical teacher becomes the method of teaching. Finally, in the clinical supervision context, the theme of *Supervisors: More Than Medical Experts* suggested that the core of medical skills and knowledge requires an embodiment that is understood in relationships as a space for learning to occur.

## Discussion

In keeping with accreditation standards for all Canadian academic departments, learner evaluations and feedback are solicited on medical educator effectiveness. An objective of the current study was to qualitatively analyze available educator evaluation data in different educational environments to inform a scholarly understanding of perceived educator effectiveness from the view of psychiatry learners. As Canadian psychiatry programs prepare to transition to the Royal College of Physicians and Surgeons of Canada’s Competence By Design (CBD) framework, there is renewed interest in faculty development.^26^ By identifying qualities of an effective educator in psychiatry, we hope to inform future directions of research specifically targeted to faculty development of medical teachers and clinical supervisors in psychiatry. The themes and sub-themes identified from the current research provide unique insights into what skills and qualities could be fostered in psychiatry educators to promote educator competence. For instance, developing and honing interpersonal, communication, and mentorship skills may promote the development of a strong educator- learner relationship. Psychiatry educators can use this relationship as a means for providing and documenting meaningful assessments and feedback. In this way, learner assessment and the provision of feedback becomes less of a monotonous process of checking boxes, and more of a personal, active engagement to promote learner outcomes within the CBD framework.^26^

Research to-date has focused on medical education in a broad sense, as opposed to skills specific to individual medical specialties. A pivotal paper in medical education by Sutkin and colleagues described qualities of good medical teachers *in general*.^15^ Categorical attributes of educators described by learners in the current study aligned with previous frameworks and did not introduce new qualities that may be considered as a core skill for medical educators in psychiatry.^15,16^ Despite the specialty- specific focus of the current study, our findings aligned with taxonomies of previously reported non- specialty-specific educator skills. The findings from the current study suggest a dynamic phenomenon in the data that goes beyond a taxonomy of educator characteristics, introducing an emotional and relational logic that was not directly referred to in the evaluation form questions. Nevertheless, it is interesting to consider the possibility that other medical specialties may require a different set of core personal characteristics.

Telio and colleagues have written about the notion of an educational alliance that forms between supervisors and learners.^18^ This concept was informed by the notion of a therapeutic alliance which is necessary for clinical work in psychotherapy, with critical elements of the relationship including a shared understanding of the work and its process, as well as the importance of the patient’s ability to like and trust their therapist. Although they suggested that there are limits to viewing the educational relationship as being in parallel to a therapeutic relationship, the findings from the current study may support using this type of framework to better explore the relational valences and tensions that are articulated by learners. Given the relative dearth of theoretical approaches to understanding educational relationships and their development in medical education, this approach would be helpful to better explore the meaning behind outlier comments that suggest a deeply emotive experience by using rich descriptions such as *“felt quite beaten up.”* It is unclear from the current study whether educational relationships described by learners are synonymous with an educational alliance. However, both ideas allude to relationships as an important skill and context for psychiatry faculty development across both medical teaching and clinical supervision domains.^2^ The centrality of an educational relationship and its potential similarities and dissonances with clinical rapport emerged as an interesting subsequent research focus and as a faculty development issue.

Tiberius and colleagues have suggested that, while there may be some valuable guiding principles, the notion of relationships in medical education can best be understood as a set of dilemmas which cannot be solved, but rather appreciated.^27^ In addition to thinking about the idea of an educational relationship and its parallels in therapeutics, the notion of the learner’s affective perceptions emerged as a significant surprise in the current study, with the narrative comments opening a very rich and meaningful discussion and potential for scholarship.

While one of our intentions was to answer the question “What do undergraduate and postgraduate medical learners perceive about educator effectiveness in psychiatry?” to inform specialty- specific faculty development efforts, the data would not cooperate with the question and instead appeared to create a new agenda. This phenomenon is consistent with normal practices of rigorous qualitative research methodology in which the research question may change as fieldwork proceeds.^20^ Surprisingly, analysis of the current data produced unique themes of educational relationships and emotional tensions within psychiatry education.

There were a number of limitations to the current study. An optimal research setting would have been to pose a question *a priori* and then construct a methodology to respond to that question. If the study had been conceptualized and then conducted, a mixed methods approach may have been more robust. However, data were available in which a qualitative methodology was the most appropriate tool for analysis. Additionally, the data were based on learner feedback without an option to correlate feedback about the quality of the educational experience with educational outcomes. Although our data provided rich information about psychiatry learners’ perceptions about what makes a good educator in psychiatry, it was not possible to determine whether these perceptions translated into better skills, knowledge, and/or patient care. There was also a paucity of constructive feedback, and this may reflect a widespread tendency to minimize conflict, even in the format of anonymous feedback forms. On the other hand, this may also be in part due to psychiatry residents’ ability to select their own clinical supervisors (which is a unique feature of McMaster University’s psychiatry residency program) and the potential relationships that have been formed throughout psychiatry training.

### Conclusion

The current paper opens possibilities for considering the importance of conceptualizing learning as an emotional situation, which would require new theoretical paradigms in medical education. While it is somewhat unsurprising that relationships and alliances matter in psychiatry, future research on specialty-specific qualities of medical educators in other specialties will allow for comparison across specialties. In particular, given that the theme of *Relationships Matter* was common to both medical teaching and clinical supervision environments in psychiatry, it would be interesting to see if this theme emerges in other medical specialties. Findings from the current study can be used to inform essential faculty development initiatives in psychiatry, particularly for early career psychiatrists developing an innovative teaching philosophy.
